# l-Fucose-containing arabinogalactan-protein in radish leaves

**DOI:** 10.1016/j.carres.2015.07.002

**Published:** 2015-10-13

**Authors:** Miho Inaba, Takuma Maruyama, Yoshihisa Yoshimi, Toshihisa Kotake, Koji Matsuoka, Tetsuo Koyama, Theodora Tryfona, Paul Dupree, Yoichi Tsumuraya

**Affiliations:** a Division of Life Science, Graduate School of Science and Engineering, Saitama University, 255 Shimo-okubo, Sakura-ku, Saitama 338-8570, Japan; b Department of Biochemistry and Molecular Biology, Faculty of Science, Saitama University, 255 Shimo-okubo, Sakura-ku, Saitama 338-8570, Japan; c Division of Material Science, Graduate School of Science and Engineering, Saitama University, 255 Shimo-okubo, Sakura-ku, Saitama 338-8570, Japan; d School of Biological Sciences, Department of Biochemistry, Cambridge University, Hopkins Building, The Downing site, Tennis Court Road, Cambridge, Cambridge CB2 1QW, UK

**Keywords:** *Raphanus sativus* L., Arabinogalactan-protein, Exo-β-(1→3)-galactanase, l-Fucose, Radish

## Abstract

The carbohydrate moieties of arabinogalactan-proteins (AGPs) have β-(1→3)-galactan backbones to which side chains of (1→6)-linked β-Gal residues are attached through *O*-6. Some of these side chains are further substituted with other sugars. We investigated the structure of l-Fuc-containing oligosaccharides released from the carbohydrate moieties of a radish leaf AGP by digestion with α-l-arabinofuranosidase, followed by exo-β-(1→3)-galactanase. We detected a series of neutral β-(1→6)-galactooligosaccharides branching variously at *O*-3 of the Gal residues, together with corresponding acidic derivatives terminating in 4-*O*-methyl-GlcA (4-Me-GlcA) or GlcA at the non-reducing terminals. In neutral oligosaccharides with degree of polymerization (dp) mainly higher than 10, l-Fuc groups were attached through l-Ara residues as the sequence, α-l-Fuc*p*-(1→2)-α-l-Ara*f*-(1→. This sequence was verified by isolation of the pentasaccharide α-l-Fuc-(1→2)-α-l-Ara*f*-(1→3)-β-Gal-(1→6)-β-Gal-(1→6)-Gal upon digestion of the higher oligosaccharides with endo-β-(1→6)-galactanase. By contrast, in lower polymerized (predominantly dp 4) acidic oligosaccharides, l-Fuc groups were attached directly at the non-reducing terminals through α-(1→2)-linkages, resulting in the release of the tetrasaccharides, α-l-Fuc*p*-(1→2)-β-GlcA-(1→6)-β-Gal-(1→6)-Gal and α-l-Fuc*p*-(1→2)-β-4-Me-GlcA-(1→6)-β-Gal-(1→6)-Gal. In long acidic oligosaccharides with dp mainly higher than 13, l-Fuc groups localized on branches were attached to the uronic acids directly and/or l-Ara residues as in the neutral oligosaccharides.

## Highlights

• A radish leaf arabinogalactan-protein (AGP) that contains l-fucose (l-Fuc) residues.
• Structure of the AGP was analyzed by digestion with exo-β-(1→3)-galactanase.
• l-Fuc found attached to both uronic acids and l-arabinose through α-(1→2)-linkages.
• Neutral and acidic l-Fuc-containing oligosaccharides from the AGP were isolated.

## Graphical abstract

**Figure:**
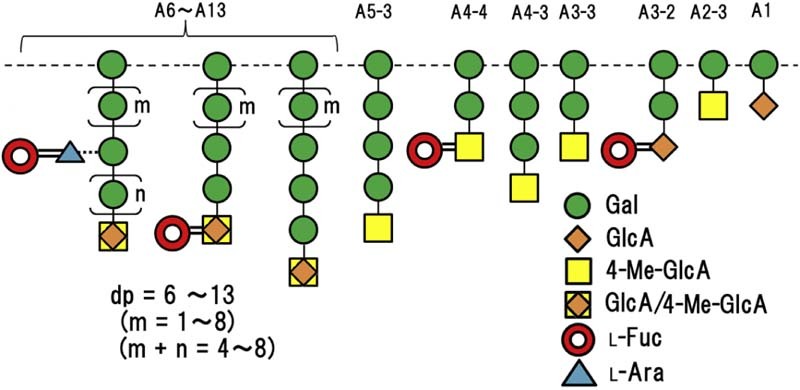

## 1 Introduction

Arabinogalactan-proteins (AGPs) are proteoglycans/glycoproteins found in cell walls, plasma membranes, and extracellular secretions of plants. They function in various aspects of plant growth and development, such as programmed cell death, somatic embryogenesis, cell expansion, and root formation and development.^1–5^ They are composed largely (>90%) of carbohydrate with some protein, typically rich in the amino acids Hyp/Pro, Ala, and Ser/Thr. The carbohydrate moieties (arabinogalactans, AGs) of AGPs consist of β-(1→3)-galactan main chains to which side chains of (1→6)-linked β-Gal residues are attached through *O*-6. The side chains are further substituted with l-Ara residues and modified with other sugar residues, such as GlcA, 4-*O*-methyl-glucuronic acid (4-Me-GlcA, abbreviated MeGlcA in the tables of this article), l-Fuc, l-Rha, and Xyl.

The presence of l-Fuc in AGPs may be considered one of the characteristic features of brassicaceous (cruciferous) plants, as it has been observed in the leaves and roots of Arabidopsis (*Arabidopsis thaliana*), leaves and primary roots of radish (*Raphanus sativus* L.), leaves of rape (*Brassica campestris* L.), and others.^6–9^ However, the sugar is also present in the AGPs of thyme (*Thymus vulgaris* L.) leaves and celery (*Apium graveolens*) seeds.^10,11^ l-Fuc residues are contained—mainly—in the sequence, α-l-Fuc*p*-(1→2)-α-l-Ara*f*-(1→, attached at *O*-3 of some Gal residues along the β-(1→6)-galactan side chains of radish and Arabidopsis leaf AGPs.^7,9^ Among various glycosyltransferases involved in the synthesis of AGs, Wu et al. have identified AtFUT4 and AtFUT6 genes encoding α-(1→2)-l-fucosyltransferases (FUTs) for Arabidopsis fucosylated AGPs, which incorporate terminal α-l-Fuc residues at the *O*-2 positions of adjacent l-Ara residues of AGPs.^12^ Recently, Tryfona et al. have reported the presence of internal l-Fuc residues substituted with Xyl residues as a sequence, α-Xyl*p*-(1→3)-α-l-Fuc*p*-(1→2)-α-l-Ara*f*-(1→, in leaf and root AGPs of Arabidopsis in addition to the above sequence when they investigated the AGs of the Arabidopsis *fut4*, *fut6*, and double *fut4/fut6* mutants.^13^ The biological roles of AGP fucosylation, however, remain unclear although there are some lines of evidence suggesting that l-Fuc may be involved in signaling and development of the plants. The biosynthesis of l-Fuc is affected in the Arabidopsis mutant *mur1* where a reduction of l-Fuc of only 40% causes a decrease of about 50% in root cell elongation. The short root phenotype is also induced by a lectin that probably recognizes α-l-Fuc*p*-(1→2)-α-l-Ara*f*-(1→ groups on AGP, suggesting that the short root phenotype of *mur1* is indeed caused by underfucosylation of AGPs.^14^ By contrast, Liang et al. and Tryfona et al. have reported that the *mur1* short root phenotype cannot be attributed entirely to the lack of l-Fuc on root AGPs, since the *fut4/fut6* mutant (lacking AGP fucosylation) did not show the root phenotype when compared to wild-type Arabidopsis.^13,15^ They postulated that the *mur1* mutant is affected by other l-Fuc-containing components, such as xyloglucan, rhamnogalacturonan-I and -II, and *N*-linked glycans, besides AGPs and proposed the involvement of fucosylated AGPs in salt sensitivity in root growth.

It has been suggested that AGPs are enzymatically processed *in planta* to release oligosaccharide signals, which play a role in physiological processes.^2,3,16^ It is thus important to determine the exact sugar sequences of AGs. In this context, we previously found several AG-specific hydrolases, such as exo-β-(1→3)-galactanase and endo-β-(1→6)-galactanase.^17,18^ Using these enzymes, we have analyzed the structure of the AG of radish mature root AGP, focusing especially on the distribution of l-Ara residues along β-(1→6)-galactan side chains of the AG.^19^ We observed that single or disaccharide l-Ara*f* units are attached at different Gal residues through *O*-3 of a series of neutral β-(1→6)-galactan side chains with degree of polymerization (dp) 1 to up to at least 19, together with corresponding acidic side chains terminating in 4-Me-GlcA at the non-reducing terminals. The high variability in length of the side chains observed in radish root AGP is consistent with structural models for AGPs from wheat (*Triticum aestivum* L.) flour and Arabidopsis leaves and roots.^9,20^ These models differ from an alternative model proposed by Tan et al. for AGPs heterologously expressed in tobacco (*Nicotiana tabacum*), which has repeating units of 15 sugar residues comprising a (1→3)-linked β-Gal backbone with internal single (1→6)-linked β-Gal “kink” residues. The backbone branches into (1→6)-linked β-Gal residues, to which l-Ara, GlcA, and l-Rha are attached.^21^

In this work, we characterize the structure of the carbohydrate moieties of a fucosylated AGP of radish leaves and find unreported sugar sequences, in which l-Fuc residues are located at non-reducing terminals attached to GlcA and 4-Me-GlcA through α-(1→2)-linkages in addition to the above α-l-Fuc*p*-(1→2)-α-l-Ara*f*-(1→ sequence. The analysis of the variable localization of l-Fuc may contribute to our understanding of the various possible roles of fucosylated AGPs.

## 2 Experimental

### 2.1 Materials

Sephadex G-100, CM-Sepharose Fast Flow, and Sephacryl S-200 were obtained from GE Healthcare Life Sciences (Tokyo, Japan). Bio-Gel P-2 (extra fine) and DEAE-cellulose were obtained from Bio-Rad Laboratories (Tokyo, Japan), and Serva Electrophoresis GmbH (Heidelberg, Germany), respectively.

Recombinant exo-β-(1→3)-galactanase (rIl1,3Gal) (EC 3.2.1.145) from *Irpex lacteus* was prepared as described previously.^17^ This enzyme specifically releases Gal from unsubstituted β-(1→3)-galactan chains in AGs, and can bypass branching points at which β-(1→6)-galactan side chains are attached, resulting in the release of the side chains as oligosaccharides.^22^ Recombinant endo-β-(1→6)-galactanase (rTv6Gal) (EC 3.2.1.164) from *Trichoderma viride*, α-l-arabinofuranosidase (rNcAraf1) (α-l-arafase; EC 3.2.1.55) from *Neurospora crassa*, and β-glucuronidase (rAnGlcAase) (β-GlcAase, EC 3.2.1.31) from *Aspergillus niger* expressed in *Pichia pastoris* were prepared as reported previously.^18,23^ The endo-β-(1→6)-galactanase has the specific capacity to release Gal, β-Gal-(1→6)-Gal, and 4-Me-β-GlcA-(1→6)-Gal as the main final reaction products from radish root AGP pretreated with α-l-arafase. The β-galactosidase (EC 3.2.1.23) (grade VIII) of *Escherichia coli* was obtained from Sigma-Aldrich Japan (Tokyo, Japan).

Powdered liver of a marine gastropod, *Charonia lampas*, was a gift from Seikagaku Corporation (Tokyo, Japan). From the liver, α-l-fucosidase (α-l-fucase; EC 3.2.1.51)^24^ was partially purified by (NH_4_)_2_SO_4_ fractionation, followed by ion-exchange chromatography on a column of CM-Sepharose Fast Flow and gel-filtration on a column of Sephacryl S-200 (data not shown). The enzyme assay was performed by incubation with 0.5 mM *p*-nitrophenyl-α-l-fucopyranoside (Sigma-Aldrich Japan) in 25 mM acetate buffer (pH 4.0) containing 0.15 M NaCl at 37 °C for a suitable time. The liberated *p*-nitrophenol was determined. Activities of the enzymes applied in this study are expressed as enzyme units (U), in which one unit is defined as the amount of each enzyme that releases 1 µmol of the corresponding product per minute.

Radish leaf AGP (designated R-II) was prepared as described previously.^7^ β-(1→6)-Galactobiose and -triose were prepared as described previously.^22^ The (oligo)saccharides β-GlcA-(1→6)-Gal and β-GlcA-(1→6)-β-Gal-(1→6)-Gal, and also 4-Me-GlcA, 4-Me-β-GlcA-(1→6)-Gal, and 4-Me-β-GlcA-(1→6)-β-Gal-(1→6)-Gal were prepared as described previously.^25^ Cyclic β-(1→2)-glucan was donated by Prof. M. Hisamatsu, Mie University and used for methylation analysis (see the next section).

### 2.2 Carbohydrate analyses

Total sugar content was determined by the phenol–sulfuric acid method.^26^ Uronic acid content was determined by a modified carbazole–sulfuric acid method^27^ using GlcA as the standard. 6-Deoxyhexose was determined by the method of Dische and Shettles^28^ using l-Fuc as the standard. Reducing sugars were determined by the method of Nelson,^29^ as modified by Somogyi,^30^ using Gal as the standard.

Paper chromatography of sugars was performed by the descending technique, using Whatman No. 1 or 3MM paper and A, 6:4:3 (v/v/v) 1-butanol/pyridine/water or B, 5:2:3 (v/v/v) l-butanol/acetic acid/water as solvent systems. Sugar spots on the paper chromatograms were visualized with an alkaline AgNO_3_ reagent. Separation of monosaccharides was carried out by high performance anion-exchange chromatography with pulsed amperometric detection (HPAEC–PAD) using a Dionex DX-500 liquid chromatograph fitted with a CarboPac PA-1 column (4 × 250 mm; Dionex Japan, Osaka, Japan) and a pulsed amperometric detector as described previously.^31^ Gas liquid chromatography (GLC) of neutral sugars as their alditol acetate derivatives was done with a Shimadzu gas chromatograph GC-6A fitted with a column (0.28 mm × 50 m) of Silar-10C or a column (0.22 mm × 25 m) of BPX70, according to the method of Albersheim et al.^32^ Methylation was performed by the Hakomori method,^33^ and the products were analyzed by GLC. The carboxyl groups of GlcA/4-Me-GlcA residues in permethylated acidic oligomers were reduced with LiAlH_4_^34^ and the resulting methylated derivatives of Glc/4-*O*-methyl-Glc were analyzed by GLC. The standard 6-*O*-acetyl-1,2,3,4,5-penta-*O*-methyl-galactitol [a reduction product of (1→6)-linked Gal residues at reducing terminals of oligosaccharides] was prepared by methylation of β-(1→6)-galactobiitol. 1,2,5-Tri-*O*-acetyl-3,4,6-tri-*O*-methyl-glucitol [a reduction product of (1→2)-linked GlcA/4-Me-GlcA residues] was also prepared by methylation of cyclic β-(1→2)-glucan. They were applied on GLC to identify methylated sugars derived from sample oligosaccharides by comparison of their retention times with those of the standards.

Matrix-assisted laser desorption/ionization time-of-flight mass spectrometry (MALDI–TOF/MS) of oligosaccharides was performed with a Bruker AutoflexIII (Bruker Daltonics, Bremen, Germany).^25^ MALDI–TOF–MS/MS was also used to examine the sequence of oligosaccharides. Linkage analysis of oligosaccharides was performed by ^13^C-NMR spectroscopy. ^13^C-NMR spectra were recorded at 100 MHz with a Bruker DRX-400 spectrometer as described previously.^19^

### 2.3 Perdeutero-methylation of oligosaccharides and MALDI–TOF/TOF–MS/MS

Perdeutero-methylation of oligosaccharides was performed by the method of Ciucanu and Kerek,^35^ using deuteromethyl iodide as described previously.^9,20^ Deutero-methylated samples were analyzed by MALDI–TOF/TOF–MS/MS (4700 Proteomics Analyzer, Applied Biosystems, Foster City, CA, USA) and high energy MALDI–collision induced dissociation (MALDI–CID) spectra were acquired with an average of 10,000 laser shots/spectrum, using a high collision energy (1 kV). The oligosaccharide ions were allowed to collide in the CID cell with argon at a pressure of 2 × 10^−6^ Torr.^9,20^

### 2.4 Enzymatic degradation of radish leaf AGP

**Fig. 1:**
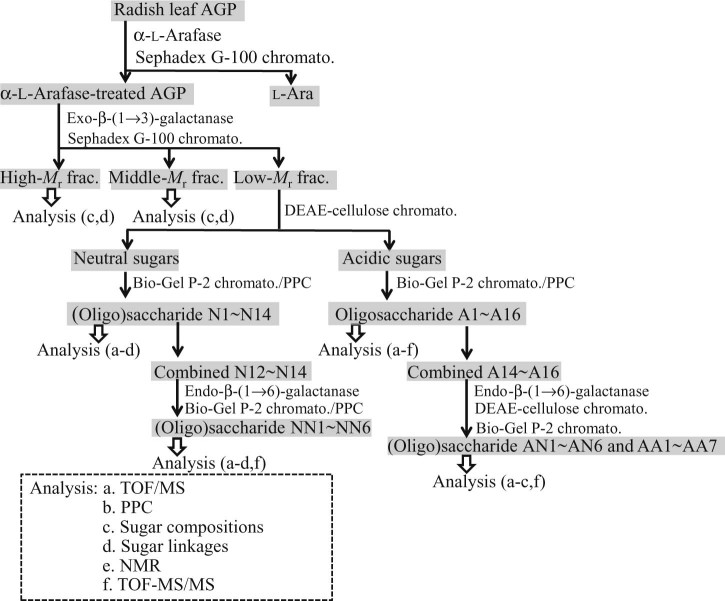
A flow diagram showing successive enzymatic fragmentation of the carbohydrate moieties of radish leaf AGP with α-l-arafase, exo-β-(1→3)-galactanase, and endo-β-(1→6)-galactanase. Fractionation and analytical methods (*a* to *e* in the *dotted box*) of the resulting oligosaccharides are also shown.

A flow diagram of successive enzymatic digestions of the carbohydrate moieties of radish leaf AGP, followed by fractionation and analytical methods of the resulting oligosaccharides, is summarized in Fig. 1. A sample (55 mg, weight basis) of radish leaf AGP was digested for 24 h at 37 °C with α-l-arafase (0.95 U) in 10 mM citrate buffer (pH 3.4, 1.8 mL). After heating in a boiling water bath for 5 min, the digestion product was chromatographed on a 2.2 × 90-cm Sephadex G-100 column equilibrated and eluted with 1% acetic acid. The elution of total sugar was monitored by the phenol–sulfuric acid method.^26^ The enzyme-modified AGP (α-l-arafase-treated AGP) eluted at high-*M*_r_ was recovered, evaporated repeatedly with water, and lyophilized (35 mg).

The modified AGP was digested with exo-β-(1→3)-galactanase (0.52 U) in 50 mM acetate buffer (pH 4.6, 0.5 mL). This digestion was repeated several times with the enzyme. The following is a typical example of the fractionation performed on the digestion products. When the liberation of reducing sugar reached a plateau at 31.8% hydrolysis (reducing sugar as Gal/total sugar), the reaction was terminated by heating for 3 min and the digest was desalted by passage through a 1.7 × 6-cm column of Dowex 50W (H^+^). The hydrolyzed sample was chromatographed on the Sephadex G-100 column equilibrated with 1% acetic acid, and fractions (each 3.0 mL) were collected. The degradation products were thus resolved into three fractions containing high- (2.2 mg), middle- (2.2 mg), and low-*M*_r_ (13.5 mg) saccharides (calculated based on total sugar content), which were pooled separately and concentrated. The high- and middle-*M*_r_ fractions were analyzed for sugar composition and type of glycosidic linkages as shown in Fig. 1. The low-*M*_r_ fraction (AG oligosaccharides) was chromatographed on a 2.6 × 8-cm DEAE-cellulose (HCO_3_^−^) column. Neutral sugars (7.0 mg; 52% of the low-*M*_r_ fraction) were eluted with water, while acidic sugars (6.4 mg; 47%) were eluted as a single peak at 50 mM, after a linear gradient (0–0.5 M, 200 mL) of NaHCO_3_. The acidic fraction was desalted on a Dowex 50W (H^+^) column. Subsequent fractionation of neutral and acidic oligomers was achieved by chromatography on a 2.6 × 90-cm Bio-Gel P-2 column, equilibrated and eluted with 1% acetic acid. Fractions (2.0 mL) were collected. Neutral sugars were resolved into 14 fractions (N1–N14; 4.5 mg; 33% of the low-*M*_r_ fraction), while acidic sugars yielded 16 fractions (A1–A16; 6.3 mg; 47%). Some selected fractions were further separated by preparative paper chromatography with solvent A (for neutral sugars) and B (for acidic sugars).

### 2.5 Digestion of oligosaccharides with glycosidases and endo-β-(1→6)-galactanase

Degradation of oligosaccharides derived from enzymatic digestion of α-l-arafase-treated AGP with exo-β-(1→3)-galactanase (see below) was carried out using several procedures in order to achieve complete and quantitative hydrolysis. The resulting hydrolysis products were subjected to HPAEC–PAD analysis. We applied chemical hydrolysis by using trifluoroacetic acid (TFA) together with combinations of sufficient amounts of α-l-fucase, β-GlcAase, α-l-arafase, β-galactosidase, and endo-β-(1→6)-galactanase depending on the sugar compositions expected from the MALDI–TOF/MS data of each oligosaccharide. Since acid hydrolysis resulted in a loss of recovery of 4-Me-GlcA, an enzymatic approach was applied here. The acidic oligosaccharide A3-2 (see the results section for preparation) (30 µg) was first hydrolyzed by heating with 2 N TFA (540 µL) for 1 h at 121 °C. After drying, the hydrolysate was then incubated with β-GlcAase (16 mU) in 10 mM acetate buffer (pH 4.6, 30 µL) for 14 h at 37 °C to ensure complete hydrolysis of remaining aldobiouronic acid (GlcA·Gal) resistant to acid hydrolysis. The reaction was terminated by boiling for 2 min. A11 (30 µg) was incubated with α-l-fucase (14 mU), β-GlcAase (10 mU), α-l-arafase (33 mU), and endo-β-(1→6)-galactanase (50 mU) in 10 mM acetate buffer (pH 4.6, 40 µL) for 17 h at 37 °C. Then β-galactosidase (34 mU) was added to the mixture, which was adjusted to final 25 mM phosphate buffer (pH 7.2, total volume 48 µL). The mixture was incubated for an additional 17 h at 37 °C. The neutral oligosaccharide N5 (30 µg) was hydrolyzed by the TFA method.

l-Fuc-containing higher neutral oligosaccharides (N12–N14; 5.1 mg) were further hydrolyzed by incubation with endo-β-(1→6)-galactanase (210 mU) in order to determine the localization of l-Fuc residues along the (1→6)-linked β-Gal side chains of radish leaf AG. The resulting (oligo)saccharides were fractionated by the Bio-Gel P-2 column as described above, and analyzed for their structure. A selected fraction was further separated by preparative paper chromatography. Similarly, l-Fuc-containing higher acidic oligosaccharides (A14–A16, 10 mg) were degraded with endo-β-(1→6)-galactanase (210 mU) with the aid of exo-β-(1→3)-galactanase (250 mU) and α-l-arafase (100 mU). The hydrolysate was fractionated into neutral (4.3 mg) and acidic (3.8 mg) oligosaccharides by a DEAE-cellulose column as above. Each oligosaccharide fraction was further fractionated on the Bio-Gel P-2 column (Fig. 1).

### 2.6 Hemagglutination inhibition assay

Hemagglutination inhibition was assayed by serial 2-fold dilution of the digestion products of radish leaf AGP with a constant amount of eel anti-H sera (lectin). The H-like activity was determined by addition of human O erythrocytes and the highest dilution factor 2*^n^* still causing complete inhibition of hemagglutination was recorded.^7^

### 2.7 Determination of protein concentration

The concentration of protein was determined by the method of Bradford^36^ with BSA as the standard.

## 3 Results

### 3.1 Enzymatic degradation of radish leaf AGP and isolation of AG oligosaccharides

**Fig. 2:**
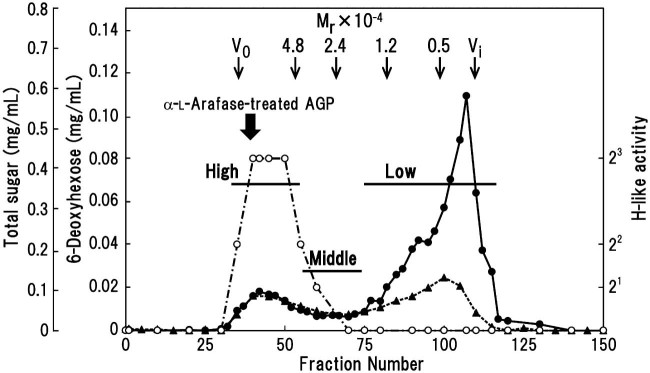
Chromatography on a Sephadex G-100 column of the exo-β-(1→3)-galactanase digested radish leaf AGP, which was pretreated with α-l-arafase. Hydrolysis products were separated into high-, middle-, and low-*M*_r_ fractions, and pooled as indicated by *bars*. The amount of total sugar (*closed circles*) and 6-deoxyhexose (l-Fuc) (*closed triangles*), and the H-like activity (*open circles*) were determined. The column was calibrated with high-*M*_r_ dextran (*V_0_*), pullulans with known *M*_r_ (Shodex Standard P-82; Showa Denko, Tokyo, Japan), and Gal (*V_i_*). The elution position of the α-l-arafase-treated AGP is also indicated.

Some properties of the radish leaf AGP are as follows: *M*_r_, 75,000 on a column of Sepharose CL-6B; Kjeldahl N, 0.7% (w/w) equivalent to a protein content of about 4.4%; sugar composition, l-Fuc:l-Ara:Gal:4-Me-GlcA:GlcA = 6:33:56:4:1 (mol%).^7^ The majority of l-Ara residues were removed by α-l-arafase digestion, and the sugar composition of the modified AGP changed to 7:9:75:6:3. The α-l-arafase-treated AGP was then hydrolyzed with exo-β-(1→3)-galactanase. The resulting products were resolved on a Sephadex G-100 column into high-, middle-, and low-*M*_r_ fractions (Fig. 2). Most (75%) of sugars in the α-l-arafase-treated AGP were apparently released as (oligo)saccharides, leaving a high-*M*_r_ fraction, possibly corresponding to the core protein still decorated with truncated AG chains. Accordingly, most uronic acid was released as oligosaccharides. The sum of the proportions of 6-deoxyhexose (l-Fuc) residues in the high- and middle-*M*_r_ fractions was almost equal to that in the low-*M*_r_ fraction, indicating a relatively high amount of the sugar in the inner parts of the AG. Even though l-Fuc existed in the low-*M*_r_ fraction, the H-like activity was detected in the high- and middle-*M*_r_ fractions, but not in the low-*M*_r_ fraction. Localization of l-Fuc in sugar sequences in both the low- and high-*M*_r_ fractions is essentially the same (see below). It is thus unclear whether the potent inhibition of hemagglutination by eel anti-H sera requires l-Fuc residues in high-*M*_r_ sugar chains.

**Fig. 3:**
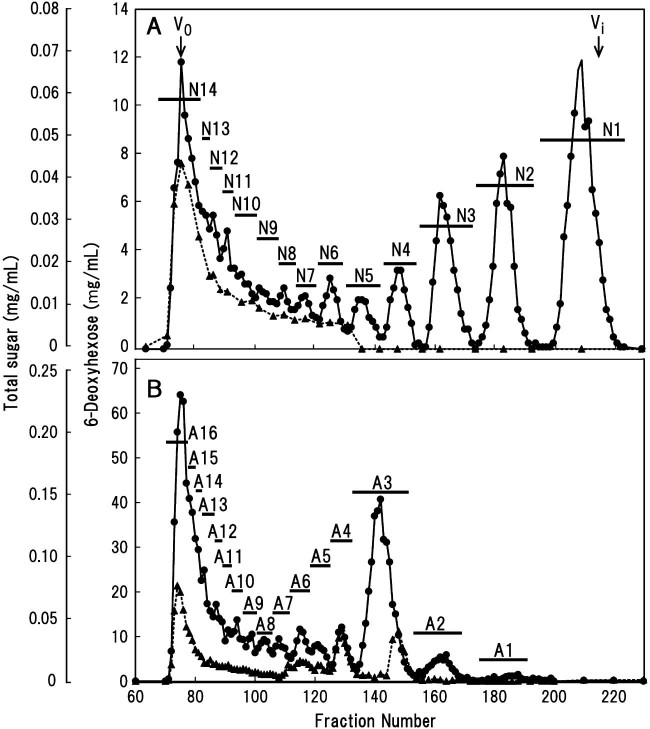
Chromatography on a Bio-Gel P-2 column of neutral and acidic sugars prepared from enzymatic digest of α-l-arafase-treated radish leaf AGP with exo-β-(1→3)-galactanase. (A) Neutral sugars were separated into N1–N14, and pooled as indicated by *bars*. (B) Acidic sugars were separated into A1–A16. The amount of total sugar (*closed circles*) and 6-deoxyhexose (l-Fuc) (*closed triangles*) was determined. The column was calibrated with high-*M*_r_ dextran (*V*_0_) and Gal (*V_i_*).

The low-*M*_r_ fraction was resolved by chromatography on a DEAE-cellulose column into two fractions containing neutral and acidic sugars in an almost equal proportion (Supplementary Fig. S1). Subsequent Bio-Gel P-2 column chromatography resolved the neutral sugars into 14 fractions (N1–N14) depending on dp ranging from Gal (N1) to higher saccharides eluting at the void volume (*V*_0_) of the column (Fig. 3A). Similarly, fractionation of the acidic sugars on the Bio-Gel P-2 column resulted in 16 fractions (Al–A16) (Fig. 3B). Based on MALDI–TOF/MS data (Tables 1 and 2), each oligosaccharide fraction with higher dp (>N5 and >A2) appeared to be a mixture of oligosaccharides with close dps and with different sugar compositions. The yields of these neutral and acidic sugars are listed in Tables 1 and 2. Some selected oligosaccharide mixtures (A2–A5) were further purified by preparative paper chromatography. For example, oligosaccharide mixture A2 separated into, at least, 3 fractions (A2-1, -2, and -3) in order of smaller relative rate of flow (*R*_f_) on the chromatogram.

Based on the substrate specificity of exo-β-(1→3)-galactanase it can be assumed that the Gal in the neutral sugar fraction derives from the β-(1→3)-galactan portion unsubstituted with β-(1→6)-galactan side chains of the AG.^17,22^ The amount of neutral sugars tends to decrease with increasing dp of the oligosaccharides, at least in the range from N1 (Gal) to around N8 (Fig. 3A and Table 1). The distribution of l-Fuc was restricted to higher neutral oligosaccharides (>N5). On the other hand, one particular fraction, A3 (a mixture of dp 3 and 4; see below), stood out as a relatively abundant, low dp, acidic oligosaccharide fraction among all oligosaccharide (Fig. 3B and Table 2). In contrast to neutral oligosaccharides, l-Fuc was present in both low dp acidic oligosaccharides (A3 and A4 fractions) and higher oligosaccharides (mainly, A14–A16).

### 3.2 Identification of oligosaccharides with low dp

The TOF/MS data and deduced sugar compositions of the oligosaccharides in the low-*M*_r_ fraction prepared from enzymatic digestion of the radish leaf AG are listed in Tables 1 and 2. Together with sugar compositions and PPC analysis (Supplementary Table S1 and Fig. S2), we identified N1, 2, 3, and 4 as Gal and β-(1→6)-galactobiose to -tetraose, respectively. These results indicate the presence of single units of Gal as minimal side chains (recovered as N2) of the AG, which are linked to the β-(1→3)-galactan backbone chains through β-(1→6)-linkages. Similarly, based on TOF/MS data, comparison with standards on paper chromatography, sugar composition analysis, and product analysis after digestion with β-GlcAase (Supplementary Table S1 and Fig. S3), A1 was identified as β-GlcA-(1→6)-Gal. After isolation by preparative paper chromatography, A2-1 and A2-3 were identified as β-GlcA-(1→6)-β-Gal-(1→6)-Gal and 4-Me-β-GlcA-(1→6)-Gal, respectively. A2-2 was a minor fraction and appeared to be a contaminant from A3-3. These data demonstrate the presence of single units of both GlcA and 4-Me-β-GlcA (recovered as A1 and A2-3) directly attached to either the non-reducing terminals or internal (1→3)-linked β-Gal residues of the backbone chains.

### 3.3 Structure of the acidic oligosaccharides

#### 3.3.1 Oligosaccharide A3

**Fig. 4:**
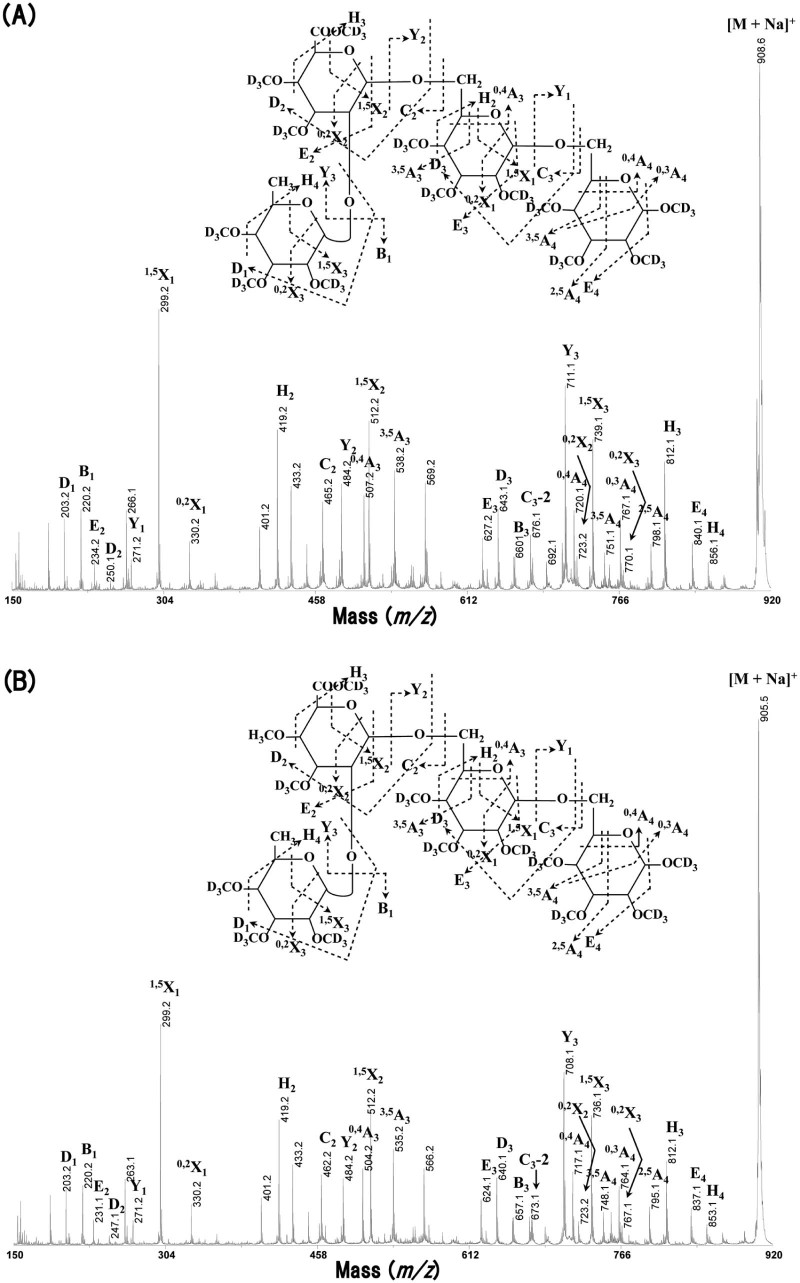
MALDI–CID of the acidic oligosaccharides (A) A3-2 and (B) A4-4.

The A3 fraction was separated into 3 oligosaccharides, A3-1, -2, and -3, by preparative paper chromatography (data not shown). A3-1 was a minor component and likely a contaminant from the A4 fraction. This component was not subjected to further analysis. From the data in Table 2 and sugar composition analysis (Supplementary Table S1), A3-2 had dp 4 and was composed of l-Fuc, GlcA, and Gal in a ratio of 1:1:2. Table 3 summarizes the glycosidic linkages of neutral and acidic oligosaccharides examined by methylation followed by GLC analysis. A3-2 appeared to contain non-reducing terminal l-Fuc*p*, *O*-2-linked Glc*p*A, internal *O*-6-linked Gal*p*, and *O*-6-linked reducing terminal Gal in approximately equimolar proportions, which suggests the structure α-l-Fuc*p*-(1→2)-β-GlcA-(1→6)-β-Gal-(1→6)-Gal. The structure of A3-2 was analyzed by MALDI–TOF/TOF–MS/MS and MALDI–CID. Fig. 4A shows the MALDI–CID spectrum for the perdeutero-methylated A3-2 (*m*/*z* 908.3). The series of Y and ^1,5^X ions indicates the sequence of the sugars on the oligosaccharide chain and shows that l-Fuc is linked to GlcA. The cross-ring fragments (^0,4^A_4_ ion, *m*/*z* 767.1 and ^0,4^A_3_ ion, *m*/*z* 507.2) show that the two Gal residues are (1→6)-linked and that the GlcA is also (1→6)-linked to the 2nd Gal from the reducing terminal, respectively. The D_2_ and E_2_ elimination ions (*m*/*z* 250.1 and *m*/*z* 234.2, respectively) and the cross-ring fragment (^0,2^X_2_ ion; *m*/*z* 723.2) show that l-Fuc is linked to *O*-2 of the GlcA.

**Fig. 5:**
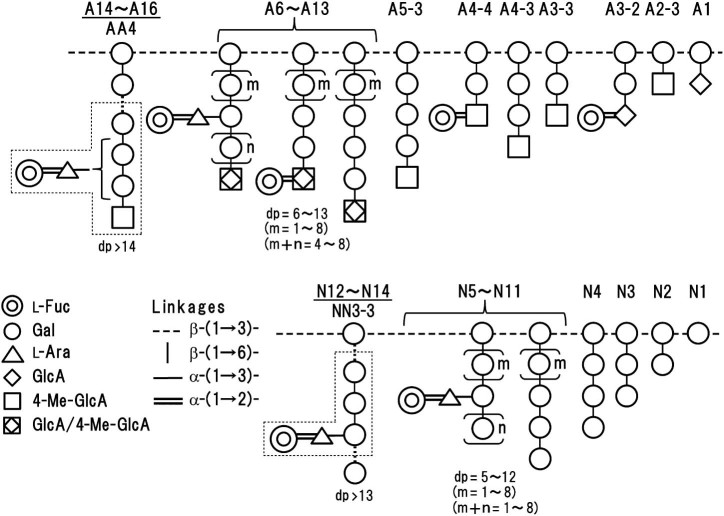
Schematic representation of the localization of l-Fuc residues along acidic (A) and neutral (B) β-(1→6)-galactooligosaccharide side chains in radish leaf α-l-arafase-treated AGP. The numbers *m*, *n* are counts of the repeating units shown in the parentheses. Sets of structures similar to A6–A13 and N5–N11 were observed in higher acidic (A14–A16) and neutral (N12–N14) oligosaccharides, respectively. Oligosaccharides liberated from the side chains by digestion with endo-β-(1→6)-galactanase are *boxed*. Note that not all side chains are depicted: for example, A2-1 is omitted.

The structure of A3-2 was further confirmed by ^13^C NMR analysis with β-GlcA-(1→6)-β-Gal-(1→6)-Gal as the reference compound. We could not achieve full assignment of carbon signals of A3-2. As shown in Table 4, the anomeric configuration of l-Fuc in A3-2 was inferred to be α from the chemical shift at 100.25 ppm, which is consistent with the previously reported value (100.22 ppm) for α-l-Fuc*p*-(1→2)-β-Gal-OMe.^37^ The anomeric configurations of GlcA and internal Gal were assigned to β. The anomeric chemical signal, 101.79 ppm, for GlcA was shifted from 103.18 ppm for the reference compound, due to substitution of its *O*-2 with l-Fuc. Taken together this allowed us to identify the structure of A3-2 as α-l-Fuc*p*-(1→2)-β-GlcA-(1→6)-β-Gal-(1→6)-Gal: see the schematic representation of the structure of the AG in Fig. 5.

According to TOF/MS (Table 2) and sugar composition analysis (Supplementary Table S1), the A3-3 structure had dp 3 and was composed of 4-Me-GlcA and Gal in a ratio of 1:2. Based on methylation analysis (Table 3) and comparison with the standard, A3-3 was identified as 4-Me-β-GlcA-(1→6)-β-Gal-(1→6)-Gal. The fact that A3-3 has the highest yield (4.8%) of the oligosaccharides among low-*M*_r_ components (Fig. 3B and Table 2) indicates that the main side chain in radish leaf AG is the disaccharide unit, 4-Me-β-GlcA-(1→6)-Gal, which has been previously observed for radish root AG modified by α-l-arafase treatment.^22^ More than half of the native radish root AG side chains are further decorated with l-Ara*f* groups as evidenced by the isolation of the l-Ara-containing acidic oligosaccharide, 4-Me-β-GlcA-(1→6)[α-l-Ara*f*-(1→3)]-β-Gal-(1→6)-Gal, by digestion with exo-β-(1→3)-galactanase.^19^ It is highly probable that such decoration with l-Ara*f* residues occurs in the case of the native radish leaf AG.

#### 3.3.2 Oligosaccharide A4

Acidic oligosaccharides other than A3 were analyzed similarly. A4 was separated into 4 fractions (A4-1, -2, -3, and -4) on paper chromatography (data not shown). A4-1 was a minor component and appeared to be a contaminant from the A5 fraction. A4-3 (dp 4) was composed of 4-Me-GlcA and Gal in a ratio of 1:3 (Table 2 and Supplementary Table S1). Based on the linkage analysis (Table 3), A4-3 was identified as 4-Me-β-GlcA-(1→6)-β-Gal-(1→6)-β-Gal-(1→6)-Gal.

A4-4 (dp 4) was composed of l-Fuc, 4-Me-GlcA, and Gal in a ratio of 1:1:2 (Table 2 and Supplementary Table S1). It appeared to contain non-reducing terminal l-Fuc*p*, *O*-2-linked 4-Me-Glc*p*A, internal *O*-6-linked Gal*p*, and *O*-6-linked reducing terminal Gal in approximately equimolar amounts (Table 3). MALDI–CID of the perdeutero-methylated A4-4 (*m*/*z* 905.3, Fig. 4B) indicates diagnostic signals for its structure like those for A3-2. In particular, the series of Y and ^1,5^X ions indicates that the order of the sugars on the oligosaccharide chain starting from the reducing terminal is: Gal, Gal, 4-Me-GlcA, and l-Fuc. The D_2_ and E_2_ elimination ions (*m*/*z* 247.1 and *m*/*z* 231.1, respectively) suggest that GlcA is methylated either at C-4 or C-6. The cross-ring fragments (^0,4^A_4_ ion, *m*/*z* 764.1 and ^0,4^A_3_ ion, *m*/*z* 504.2) are indicative of a (1→6)-linkage between the reducing end Gal and the 2nd Gal and also between the 4-Me-GlcA residue and the 2nd Gal. Finally, the cross-ring fragment (^0,2^X_2_ ion; *m*/*z* 723.2) suggests that l-Fuc is linked to *O*-2 of 4-Me-GlcA. The structure of A4-4 was hence identified as α-l-Fuc*p*-(1→2)-4-Me-β-GlcA-(1→6)-β-Gal-(1→6)-Gal (Fig. 5). These results indicate that α-l-Fuc*p* residues occur attached not only to GlcA residues (A3-2) but also to 4-Me-GlcA (A4-4) through α-(1→2)-linkages in radish leaf AG.

#### 3.3.3 Oligosaccharide A5

On paper chromatography, fraction A5 separated into at least 3 fractions (A5-1, -2, and -3) (data not shown). A5-1 and -2 were minor components and were not examined further. The data in Table 2 and sugar composition analysis (Supplementary Table S1) implied that A5-3 (dp 5) was composed of 4-Me-GlcA and Gal in a ratio of 1:4. The oligosaccharide was identified as 4-Me-β-GlcA-(1→6)-β-Gal-(1→6)-β-Gal-(1→6)-β-Gal-(1→6)-Gal.

#### 3.3.4 Oligosaccharides A6–A13

Oligosaccharides A6–A13 were analyzed by TOF/MS, subjected to sugar composition analysis, and linkage analysis (Table 3). These fractions were mixtures of oligosaccharides and appeared to consist mainly of [4-Me-GlcA, Gal], and/or [l-Fuc, 4-Me-GlcA, Gal], together with [l-Fuc, GlcA, Gal] and/or [l-Fuc, l-Ara, GlcA, Gal] in the case of A9–A13 (Table 2, see also footnote). The sugar composition analysis showed that all these fractions contained l-Fuc, l-Ara, 4-Me-GlcA, GlcA, and Gal, and the proportions (mol% ranges) were 5–10, 5–9, 8–14, 2–4, and 66–78, respectively (Supplementary Table S1). However, these data are not completely consistent with those obtained by TOF/MS, in which l-Ara-containing oligosaccharides were not detected as main signals for A6–A8. The contents of l-Fuc and l-Ara were small, but tended to increase with increasing dp of oligosaccharides (Fig. 3B and Supplementary Table S1). The data suggest that l-Fuc residues are attached to relatively long (1→6)-linked β-Gal side chains (4–11 Gal units) either directly onto non-reducing terminal GlcA/4-Me-GlcA (like A3-2 and A4-4) or through l-Ara as the sequence, α-l-Fuc*p*-(1→2)-α-l-Ara*f*-(1→, as observed in our previous study.^7^ Linkage analysis of A6 and A7 supports these structural characteristics. A6 appeared to involve non-reducing terminal l-Fuc*p* attached to *O*-2 of Glc*p*A/4-Me-Glc*p*A (Table 3). The presence of an excess amount of non-reducing terminal Glc*p*A/4-Me-Glc*p*A indicates that fucosylation occurs only on a part of the uronic acids. A7 likely contains a mixture of structures: l-Fuc*p* attached to *O*-2 of both Glc*p*A/4-Me-Glc*p*A and l-Ara*f* residues in the same, or possibly different oligosaccharide molecules.

#### 3.3.5 Oligosaccharides A14–A16

A14–A16 were derived from long acidic side chains of leaf AG, and they were mixtures of oligosaccharides composed of l-Fuc, l-Ara, 4-Me-GlcA, GlcA, and Gal. In order to determine the localization of l-Fuc along the side chains, the combined fraction (10 mg) was treated with endo-β-(1→6)-galactanase in the presence of exo-β-(1→3)-galactanase and α-l-arafase to ensure complete fragmentation of the side chains. The resulting hydrolysate was separated by ion-exchange chromatography into neutral and acidic (oligo)saccharides in approximately equal amounts (data not shown). The oligosaccharides were further separated by gel-filtration on a Bio-Gel P-2 column. Six neutral mono- and oligosaccharide fractions, AN1–AN6 (in order of increasing dp) and 7 acidic oligosaccharide fractions, AA1–AA7, were obtained (Table 5). These fractions were analyzed for their structure mainly by MALDI–TOF/MS and TOF–MS/MS.

The main acidic oligosaccharides liberated by digestion with endo-β-(1→6)-galactanase were β-GlcA-(1→6)-Gal and 4-Me-β-GlcA-(1→6)-Gal (data not shown). Liberation of these disaccharides was consistent with the mode of action of the enzyme described in Section 2. Small amounts of l-Fuc-containing acidic tetrasaccharides (AA2 and AA3) were detected, and their structures appeared to be the same as those for A3-2 and A4-4 described above. TOF–MS/MS analysis of the main signal (*m*/*z* 995.3) of AA4 (Table 5 and Supplementary Table S1) showed diagnostic fragment masses, indicative of the sequence, [l-Ara-Gal-Gal-Gal], [l-Fuc-l-Ara-Gal-Gal], and [l-Fuc-l-Ara-Gal-Gal-Gal]. Taken together, this leads to the conclusion that the main oligosaccharide in AA4 is 4-Me-β-GlcA-(1→6)[α-l-Fuc-(1→2)-α-l-Ara*f*-(1→3)]-β-Gal-(1→6)-β-Gal-(1→6)-Gal or 4-Me-β-GlcA-(1→6)-β-Gal-(1→6)[α-l-Fuc-(1→2)-α-l-Ara*f*-(1→3)]-β-Gal-(1→6)-Gal (see Fig. 5). We surmise that l-Fuc residues localize on longer fragments (AA5).

Meanwhile, neutral (oligo)saccharides liberated from acidic side chains apparently derived from the inner parts of the chains. Main fragments were Gal together with small amounts of l-Ara and β-Gal-(1→6)-Gal (data not shown). l-Fuc residues appeared to attach to the chains through l-Ara (AN3 and AN4) or l-Ara disaccharide units (AN5) (Table 5). These results indicate that l-Fuc residues in long acidic side chains of leaf AG are attached mainly to β-Gal residues either near the non-reducing terminal GlcA/4-Me-GlcA residues or inner chains through l-Ara, possibly as the sequence, α-l-Fuc*p*-(1→2)-α-l-Ara*f*-(1→, as proposed for the structures of neutral side chains (N12–N14, see below). Only a small amount of l-Fuc residues is directly attached to non-reducing terminal Glc*p*A/4-Me-Glc*p*A residues of the side chains.

### 3.4 Structure of the neutral oligosaccharides

#### 3.4.1 Oligosaccharides N5–N11

The structures of neutral oligosaccharides (N5–N11) derived from leaf AG by digestion with exo-β-(1→3)-galactanase were examined by MALDI–TOF/MS (Table 1), sugar composition analysis (Supplementary Table S1), and linkage analysis (Table 3). These fractions were mixtures of oligosaccharides composed of l-Fuc, l-Ara, and Gal, and their proportions (mol% ranges) were 5–7, 4–6, and 87–91, respectively. The content of l-Fuc and l-Ara showed a tendency to increase with increasing dp of oligosaccharides as observed for acidic oligosaccharides A6–A13 described above. TOF/MS analysis indicated that each fraction involved β-(1→6)-galactooligosaccharides with dp 5–12 and [l-Fuc-l-Ara-Gal_3–10_]. Since the proportions of l-Fuc and l-Ara were almost equal throughout the oligosaccharides, involvement of [l-Ara-Gal_4–11_] suggested by the TOF/MS data (Table 1) may probably be excluded. Linkage analyses of N5, N6, and N7 (Table 3) supports these structural characteristics, showing the presence, possibly, of single or multiple units of α-l-Fuc*p*-(1→2)-α-l-Ara*f*-(1→ attached to *O*-3 of β-(1→6)-galactooligosaccharides.

#### 3.4.2 Oligosaccharides N12–N14

N12–N14 were mixtures of higher oligosaccharides. The combined fraction (5.1 mg) was digested with endo-β-(1→6)-galactanase. The hydrolysate was separated on a Bio-Gel P-2 column, and examined for the localization of l-Fuc in fragments as for oligosaccharides A14–A16 described above. The elution profile was almost the same as that obtained for the neutral fraction (AN1–AN6) derived from the acidic side chains. In total 6 fractions NN1–NN6 were obtained (Table 5). Besides Gal and β-Gal-(1→6)-Gal as the main fragments, l-Fuc was detected in all fractions. Among these fractions, NN3 was further separated into 3 fractions, NN3-1, -2, and -3, by preparative paper chromatography. NN3-3 (dp 5) appeared to consist of l-Fuc, l-Ara, and Gal in a ratio of 1:1:3 (Supplementary Table S1). TOF–MS/MS analysis of its signal (*m*/*z* 805.3) (Table 5) showed diagnostic fragment masses, indicative of the sequences [Gal-Gal], [Gal-Gal-Gal], [l-Ara-Gal-Gal-Gal], and [l-Fuc-l-Ara-Gal-Gal-Gal]. Based on these data and the linkage analysis (Table 3), NN3-3 was identified as α-l-Fuc-(1→2)-α-l-Ara*f*-(1→3)-β-Gal-(1→6)-β-Gal-(1→6)-Gal (Fig. 5), as postulated for the neutral oligosaccharide (AN3 and AN4) derived from acidic side chains. This oligosaccharide has previously been identified in enzymatic digests of Arabidopsis leaf and root AGPs with exo-β-(1→3)-galactanase.^9,13^

### 3.5 Middle- and high-*M*_r_ fractions

Middle- and high-*M*_r_ fractions recovered after digestion of radish leaf AGP with exo-β-(1→3)-galactanase, followed by separation on a Sephadex G-100 column (Fig. 2), were examined for their sugar composition and mode of glycosidic linkage. The middle- and high-*M*_r_ fractions were composed of l-Fuc, l-Ara, Gal, 4-Me-GlcA, and GlcA in proportions of 14:14:64:6:2 and 14:18:57:9:2 mol%, respectively (Supplementary Table S1). It is likely that the high-*M*_r_ fraction is located at the inner portion of the AG attached to the core polypeptide, impeding enzyme access. The elevated proportion of l-Fuc in comparison with that (7 mol%) in α-l-arafase-treated AGP suggests that the sugar located preferentially close to the polypeptide chain of the AGP. Linkage analysis indicated that these fractions were derive from Ara*f*1→:Fuc*p*1→:→2Ara*f*1→:(4-Me-)GlcA1→:Gal1→:→2(4-Me-)GlcA1→:→3Gal1→:→6Gal1→:→3,6Gal1→ = 3:9:9:4:6:1:8:40:20 (middle) and 3:11:9:7:8:3:8:30:21 mol% (high). These data suggest that l-Fuc residues are attached mainly to *O*-2 of l-Ara*f* residues, together with Glc*p*A/4-Me-Glc*p*A residues in the high- and middle-*M*_r_ fractions, as observed for long acidic oligosaccharides (A14–A16).

## 4 Discussion

We investigated the structure of the carbohydrate moieties in a radish leaf AGP, focusing in particular on the localization of l-Fuc residues, by digesting the AGP with α-l-arafase, then exo-β-(1→3)-galactanase. The exo-β-(1→3)-galactanase enzyme released the majority (about 75%) of sugars into a low-*M*_r_ fraction as identified by gel-filtration chromatography (Fig. 2). The detection of a series of neutral and acidic side chains with increasing dps from radish leaf AGP is consistent with our previous observations concerning the structure of radish root and Arabidopsis AGPs.^9,19^ Approximately half of l-Fuc was found in the low-*M*_r_ fraction, and the remaining half was in the high-*M*_r_ fraction, indicating that l-Fuc preferentially localizes close to the core polypeptide chains. The sugar in the low-*M*_r_ fraction was further fractionated into neutral and acidic oligosaccharides in approximately equal proportion (Supplementary Fig. S1). Among the neutral (oligo)saccharides, l-Fuc was restricted mainly to higher oligosaccharides, whereas the sugar localized to lower (mainly dp 4) and higher acidic oligosaccharides in roughly equal proportion (Fig. 3). Subsequent analyses of l-Fuc-containing oligosaccharides demonstrated that the sugar localizes as α-l-Fuc*p*-(1→2)-α-l-Ara*f*-(1→ sequences attached onto *O*-3 of certain Gal residues of neutral β-(1→6)-galactooligosaccharide side chains. In contrast, the sugar localizes to non-reducing terminal 4-Me-GlcA/GlcA residues directly through α-(1→2)-linkages mainly in short (two Gal unit long) acidic side chains. In long acidic side chains, l-Fuc localizes as branches attached mainly to *O*-2 of l-Ara*f* residues, a structure similar to that found in higher neutral oligosaccharides. A minor portion of the sugar seems to be attached directly to uronic acids. The characteristic localization of l-Fuc was verified by identification of the oligosaccharides, α-l-Fuc*p*-(1→2)-β-GlcA-(1→6)-β-Gal-(1→6)-Gal (A3-2) and α-l-Fuc*p*-(1→2)-4-Me-β-GlcA-(1→6)-β-Gal-(1→6)-Gal (A4-4), as well as α-l-Fuc-(1→2)-α-l-Ara*f*-(1→3)-β-Gal-(1→6)-β-Gal-(1→6)-Gal (NN3-3) upon digestion of the higher neutral oligosaccharides with endo-β-(1→6)-galactanase (Fig. 5).

AGP genes are expressed in an organ- and tissue-specific manner as exemplified by differential expression patterns of Arabidopsis AGP-1, -9, -12, -15, and -23.^38^ In addition, AGPs and oligosaccharides released from AGPs are thought to function as physiological signaling molecules.^1–5,16^ Even though the content of l-Fuc groups in radish leaf AGP is low (6 mol%), organ-specific expression of fucosylated AGPs in primary roots and mature leaves, but not in mature roots,^8^ together with the restriction of l-Fuc groups to particular types of side chains revealed in this study, may enable fucosylated AGPs to act as signaling molecules involved in various physiological phenomena. They may, for example, play a role in regulatory interactions between l-Fuc-containing AGPs and other wall components.^15^ In this scenario it is of interest to determine how the biosynthesis of these different fucosylated oligosaccharide sequences in AGPs is regulated. Although direct attachment of l-Fuc groups to uronic acids in Arabidopsis AGPs has not been found, Wu et al. have reported that the two FUT enzymes AtFUT4 and AtFUT6 may not be functionally redundant because they differentially fucosylate particular AGPs, suggesting different physiological roles for the two enzymes.^12^ Tryfona et al., however, have reported that the two enzymes are at least partially redundant.^13^ It remains to find out whether the incorporation of l-Fuc in fucosylated AGPs is catalyzed by a single FUT or different FUTs recognizing particular acceptor sugars (i.e., l-Ara or uronic acids) in concerted action with other glycosyltransferases.

l-Rha, another 6-deoxyhexose found in AGPs beside l-Fuc, is known to occasionally localize at non-reducing terminals attached to GlcA as the sequence, α-l-Rha*p*-(1→4)-β-Glc*p*A-(1→6)-β-Gal*p*-(1→, which is found in AGPs from acacia (*Acacia Senegal*) gum and those heterologously expressed in tobacco.^21,39^ However, almost nothing is known about its physiological role and biosynthesis, which may be catalyzed by an α-(1→4)-l-rhamnosyltransferase not yet identified. The wide distribution of fucosylated AGPs among cruciferous plants^6–9^ and the distinctive localization of l-Fuc presented in this study suggest specific, though as yet undetermined, roles for l-Fuc in development of the plants.

## Figures and Tables

**Table 1:** Yields and TOF/MS data of neutral oligosaccharides separated from exo-β-(1→3)-galactanase digested α-l-arafase-treated radish leaf AGP

Fraction	Yield^a^ (%)	TOF/MS (*m*/*z*)		
Observed^b^	Calculated^c^	Sugar composition^d^
N1	8.2			Gal
N2	4.0	365.4	365.3	2Gal
N3	3.4	527.3	527.4	3Gal
N4	1.5	689.2	689.6	4Gal
N5	1.1	805.3	805.7	Fuc·Ara·3Gal
		821.3	821.7	Ara·4Gal
			821.8*	Fuc·Ara·3Gal
		851.3	851.7	5Gal
N6	1.2	967.3	967.8	Fuc·Ara·4Gal
		983.3	983.8	Ara·5Gal
			983.9*	Fuc·Ara·4Gal
		1013.4	1013.9	6Gal
N7	1.0	1129.3	1130.0	Fuc·Ara·5Gal
		1145.3	1146.0	Ara·6Gal
			1146.1*	Fuc·Ara·5Gal
		1175.4	1176.0	7Gal
N8	0.9	1307.4		
		1337.4		
N9	1.1	1469.6		
		1499.6		
N10	1.2	1631.5		
		1661.6		
N11–N13	4.4			
N14	5.3			
Total	33.3			

a Yields are calculated by dividing the sugar content of each fraction (obtained by the phenol–H_2_SO_4_ method) by the amount (13.5 mg) of the low-*M*_r_ fraction recovered after Sephadex G-100 column chromatography.

b Mass values (*m*/*z*) observed are listed for oligosaccharides N2–N10 only .

c Calculated mass values for [M + Na] ^+^ are listed. *Asterisks* indicate [M + K]^+^ values. Note that, for example, the observed mass value (983.3) for N6 may represent [Ara·5Gal + Na]^+^ and/or [Fuc·Ara·4Gal + K]^+^, which cannot be distinguished because their mass values are too close.

d Based on observed mass values, N7–N10 appear to contain several oligosaccharides, like N5 and N6, of Ara·*m*Gal (*m* = 6–9), Fuc·Ara·*n*Gal (*n* = 5–8), and *j*Gal (*j* = 7–10). N11 is a mixture of two sets of oligosaccharides with one and two Gal units more.

**Table 2:** Yields and TOF/MS data of acidic oligosaccharides separated from exo-β-(1→3)-galactanase digested α-l-arafase-treated radish leaf AGP

Fraction	Yield^a^ (%)	PPC^b^	Yield^a^ (%)	TOF/MS (*m*/*z*)		
Observed^c^	Calculated^d^	Sugar composition^e^
A1	0.5					GlcA·Gal
A2		A2-1	0.7	541.1	541.4	GlcA·2Gal
		A2-2	<0.1			
		A2-3	0.7	392.9	393.3	MeGlcA·Gal
A3		A3-1	0.3	—f		
		A3-2	0.8	687.0	687.6	Fuc·GlcA·2Gal
		A3-3	4.8	555.6	555.5	MeGlcA·2Gal
A4		A4-1	0.3	—		
		A4-2	0.2	879.0	879.7	MeGlcA·4Gal
		A4-3	0.9	716.9	717.6	MeGlcA·3Gal
		A4-4	0.8	700.9	701.6	Fuc·MeGlcA·2Gal
A5		A5-1	0.4	—		
		A5-2	0.3	—		
		A5-3	0.7	879.3	879.7	MeGlcA·4Gal
A6	1.4			1011.3	1011.8	Fuc·GlcA·4Gal
				1041.3	1041.9	MeGlcA·5Gal
					1042.0*	Fuc·MeGlcA·4Gal
A7	2.0			1173.3	1174.0	Fuc·GlcA·5Gal
				1203.3	1204.0	MeGlcA·6Gal
					1204.1*	Fuc·MeGlcA·5Gal
A8	2.3					
A9–A13	6.4					
A14	4.0					
A15	3.8					
A16	9.0					
Total	40.3					

a See the legend of Table 1.

b PPC means preparative paper chromatography.

c Mass values (*m*/*z*) observed for oligosaccharides A1–A7 only are listed.

d Calculated mass values for [M + Na] ^+^ are listed. *Asterisks* indicate [M + K]^+^ values. Note that, for example, the observed mass (1041.3) for A6 may indicate either [MeGlcA·5Gal + Na]^+^ or [Fuc·MeGlcA·4Gal + K]^+^, or both, as they cannot be distinguished due to the proximity of their mass values.

e Based on observed mass values, A6–A13 appear to be mixtures of oligosaccharides of MeGlcA·*m*Gal (*m* = 5–12) and/or Fuc·(Me)GlcA·*n*Gal (*n* = 4–11). In addition, for A9–A13, signals for Fuc·GlcA·*j*Gal (*j* = 7–11) and/or Fuc·Ara·(Me)GlcA·*k*Gal (*k* = 6–10) are also detected.

f Not determined.

**Table 3:** (A) Glycosidic linkages of neutral oligosaccharides. (B) Glycosidic linkages of acidic oligosaccharides

(A)				
Mode of glycosidic linkage^a^ (molar ratio)	N5	N6	N7	N12–N14
NN3-3
*t*-Ara*f*→	—c	0.7	0.7	—
→6Galol^b^	1.0	1.2	2.0	0.9
*t*-Fuc*p*→	1.0	1.0	1.0	1.0
→2Ara*f*1→	+^d^	0.6	0.6	0.9
*t*-Gal→	11.8	10.2	6.3	—
→3Gal1→	0.5	1.1	0.6	1.3
→6Gal1→	37.4	44.1	29.7	1.3
→3,6Gal1→	0.7	1.4	1.2	—

(B)						
Mode of glycosidic linkage^a^ (molar ratio)	A3		A4		A6	A7
A3-2	A3-3	A4-3	A4-4		
*t*-Ara*f*→	—c	—	—	—	—	5.0
→6Galol^b^	1.2	1.2	0.9	1.4	3.9	2.3
*t*-Fuc*p*→	0.9	—	—	1.0	1.0	1.0
→2Ara*f*1→	—	—	—	—	—	0.3
*t*-(Me)GlcA→	—	0.8	1.0	—	4.9	3.7
→2(Me)GlcA1→	0.9	—	—	0.8	0.7	0.2
→3Gal1→	—	—	—	—	1.1	—
→6Gal1→	1.0	1.0	2.1	1.0	17.6	18.2
→3,6Gal1→	—	—	—	—	0.6	1.4

a Samples were methylated after reduction of their reducing ends with NaBH_4_. Methyl esters of the methylated acidic oligosaccharides were reduced with LiAlH_4_ and remethylated. The resulting methylated derivatives of either GlcA or 4-Me-GlcA were analyzed as shown in (Me)GlcA. The ‘Mode of glycosidic linkage’ column is arranged in order of retention time of each methylated sugar on GLC. For neutral oligosaccharides, the molar ratio is expressed based on non-reducing terminal Fuc taken as 1.0. For acidic oligosaccharides, either *O*-6 linked Gal, non-reducing terminal (4-Me-)GlcA, or non-reducing terminal Fuc is taken as 1.0. *t*- indicates non-reducing terminal.

b *O*-6-Linked galactitol as a reduction product of (1→6)-linked reducing terminal Gal.

c Not detected or small amount.

d A small amount.

**Table 4:** ^13^C NMR data of the acidic oligosaccharide A3-2.

	Chemical shift (ppm)^a^			
l-Fuc	GlcA	GalII	GalI
β-GlcA-(1→6)-β-Gal-(1→6)-Gal^b^				
C-1 (α)				92.81
C-1 (β)		103.18	103.61	96.86
A3-2				
C-1 (α)	100.25			92.84
C-1 (β)	101.79	103.50	96.90

a Chemical shifts are based on MeOD as the reference (δ 49.5 ppm). GalI and GalII indicate reducing terminal Gal and penultimate Gal groups, respectively.

b Reference oligosaccharide.

**Table 5:** Yields and TOF/MS data of neutral and acidic fragments derived from oligosaccharides A14–A16 and N12–N14 by digestion with endo-β-(1→6)-galactanase

Fraction	Degraded products	Yield^a^ (%)	TOF/MS (*m*/*z*)		
Observed^b^	Calculated^c^	Sugar composition
A14–A16	Neutral				
AN1	11			Gal, Ara
AN2	22			2Gal
AN3	4	805.2	805.7	Fuc·Ara·3Gal
AN4	3	967.3	967.8	Fuc·Ara·4Gal
AN5	2	1261.4	1262.1	Fuc·2Ara·5Gal
AN6	1	—d		—
Acidic				
AA1	13			GlcA·Gal, MeGlcA·Gal
AA2	0.5	687.1	687.6	Fuc·GlcA·2Gal
AA3	0.5	701.1	701.6	Fuc·MeGlcA·2Gal
AA4	3	995.3	995.8	Fuc·Ara·MeGlcA·3Gal
		1011.3	1011.8	Fuc·GlcA·4Gal
AA5	2	1613.5	1614.4	2Fuc·Ara·GlcA·6Gal
			1614.4	Fuc·2Ara·MeGlcA·6Gal
AA6	1	—		
AA7	3	—		
Total		66			
N12–N14	NN1	15			Gal
NN2	40			2Gal
NN3				
NN3-1	3	967.3	967.8	Fuc·Ara·4Gal
NN3-2	1	821.3	821.7	Ara·4Gal
NN3-3	6	805.3	805.7	Fuc·Ara·3Gal
NN4	4	967.3	967.8	Fuc·Ara·4Gal
NN5	7	1261.4	1262.1	Fuc·2Ara·5Gal
NN6	7	—		
Total		83			

a Yields are calculated from the sugar content (the phenol–H_2_SO_4_ method) of each product divided by the amount (10 mg) of A14–A16 or that (5.1 mg) for N12–N14, which are taken as 100%, respectively.

b Only diagnostic mass values (*m*/*z*) observed for oligosaccharides are listed.

c Calculated mass values for [M + Na] ^+^ are listed.

d Not determined.
