# Supplementary material for: l-Fucose-containing arabinogalactan-protein in radish leaves
Source: Carbohydr Res. 2015 Oct 13;415:1–11. doi: 10.1016/j.carres.2015.07.002 (PMC4610949; doi:10.1016/j.carres.2015.07.002)
Supplement: Appendix S1 — Figs. S1–S3 and Table S1. [file mmc1.pdf]

Supplementary Information to:

L-Fucose-containing arabinogalactan-protein in radish leaves

Miho Inaba, Takuma Maruyama, Yoshihisa Yoshimi, Toshihisa Kotake, Koji Matsuoka, Tetsuo Koyama, Theodora Tryfona, Paul Dupree, Yoichi Tsumuraya*

*Corresponding author

Division of Life Science, Graduate School of Science and Engineering, Saitama University,

255 Shimo-okubo, Sakura-ku, Saitama 338-8570, Japan

E-mail, tsumura@mail.saitama-u.ac.jp, Tel, +81-48-858-3401

Supplementary Table S1.

Sugar compositions of oligosaccharide fractions obtained by enzymatic fragmentation of α-L-Arafase-treated radish leaf AGP by exo-β-(1→3)-galactanase. The analyses were performed by HPAEC-PAD as describe in the section 2.5 in "Experimental". NN3-3 and AA4 fractions were obtained by digestion of combined N12~N14 and A14~A16 fractions, respectively, by endo-β-(1→6)-galactanase.

Fraction	Sugar composition (mole % or molar ratio)				
	L-Fuc	L-Ara	Gal	4-Me-GlcA	GlcA
N1~N4	-[a]	-	100	-	-
N5	5	4	91	-	-
N6	5	4	91	-	-
N7	6	5	89	-	-
N8	7	5	88	-	-
N9	6	5	89	-	-
N10	6	5	89	-	-
N11	7	6	87	-	-
NN3-3	1.0	1.0	2.7	-	-
A1	-	-	1.0		1.0
A2-1	-	-	2.0		1.0
A2-3	-	-	1.0	1.0	-
A3-2	1.0	-	1.8		1.0
A3-3	-	-	2.1	1.0	-
A4-3	-	-	3.3	1.0	-
A4-4	1.2	-	2.2	1.0	-
A5-3	-	-	4.4	1.0	-
A6	6	5	72	14	3
A7	5	5	75	13	2
A8	5	5	77	11	2
A9	5	5	78	10	2
A10	5	6	78	9	2
A11	6	6	78	8	2
A12	6	6	78	8	2
A13	10	9	66	11	4
AA4	1.0	1.0	3.0	1.0	-
Middle-M_r	14	14	64	6	2
High-M_r	14	18	57	9	2

[a] Not detected.

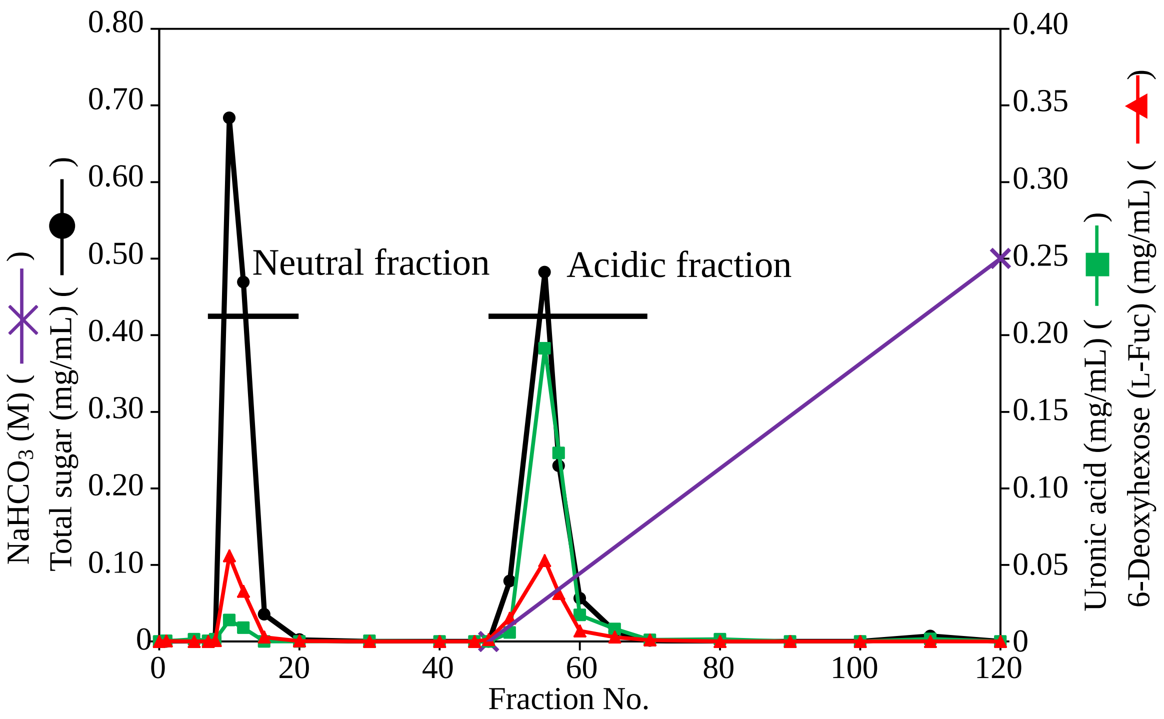

Supplementary Figure S1.

Separation by DEAE-cellulose column chromatography of neutral and acidic sugars in the low-M_r fraction obtained by digestion of α-L-Arafase-treated radish leaf AGP with exo-β-(1→3)-galactanase. The chromatography was performed as described in the section 2.4 in “Experimental”. The *bars* indicate the fractions pooled.

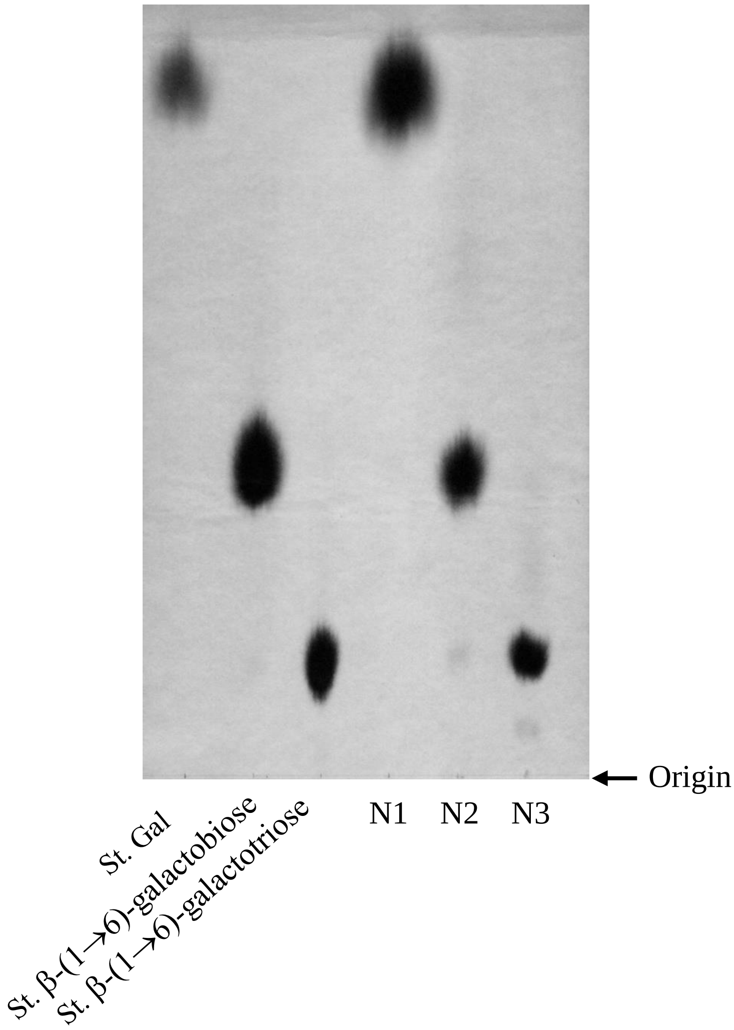

Supplementary Figure S2.

Paper chromatographic separation of N1~N3 fractions obtained by enzymatic fragmentation of α-L-Arafase-treated radish leaf AGP with exo-β-(1→3)-galactanase. The chromatography was performed with solvent A as described in the section 2.2 in "Experimental". St. means standard sugar.

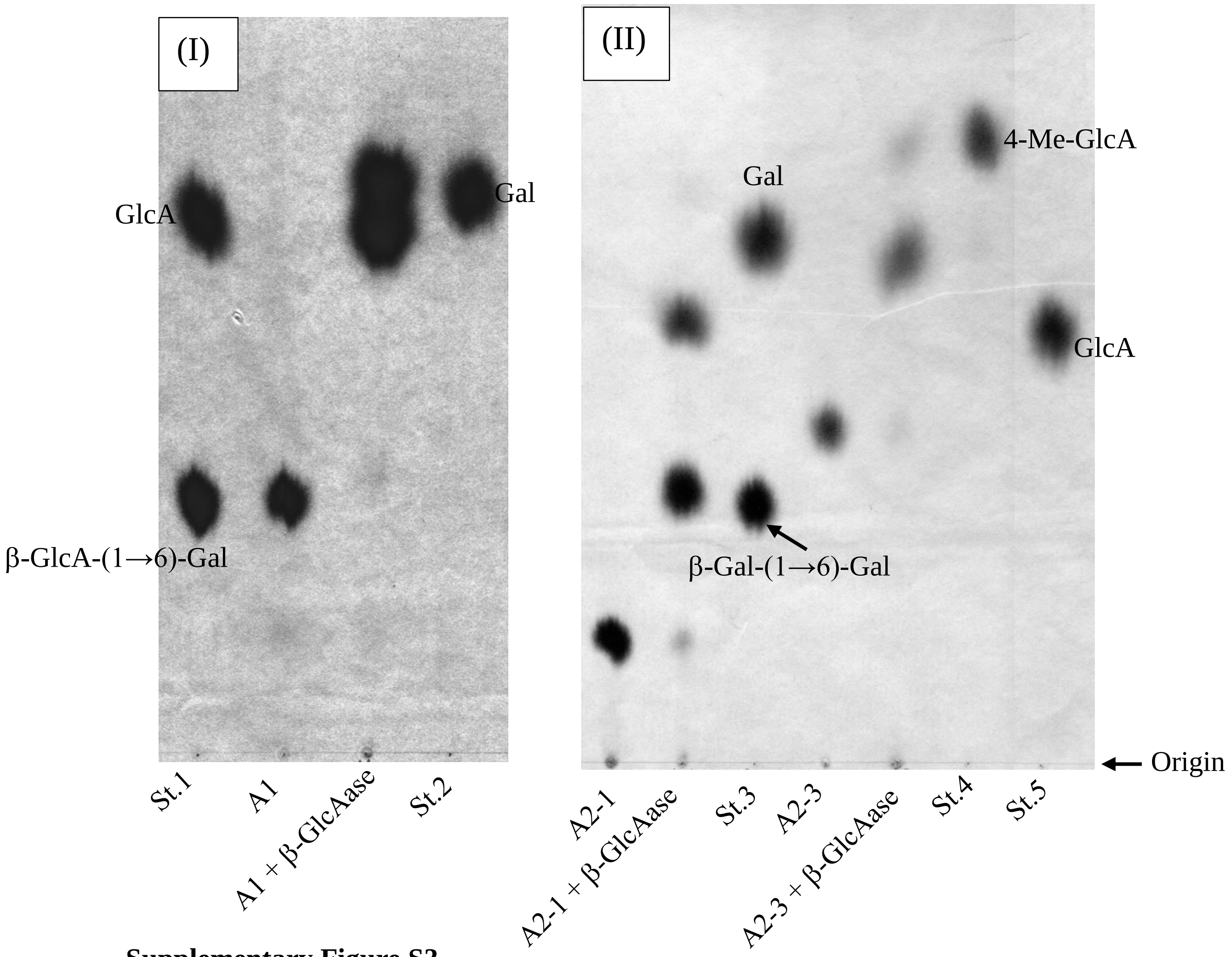

Supplementary Figure S3.

Paper chromatographic separation of A1 (panel I), and A2-1 and A2-3 (panel II) obtained by enzymatic fragmentation of α-L-Arafase-treated radish leaf AGP with exo-β-(1→3)-galactanase. The chromatography was performed with solvent B as described in the section 2.2 in "Experimental". The oligosaccharides were analyzed for their sugar compositions by digestion with β-GlcAase as described in the section 2.5.
